# A Chemosensory Protein *BtabCSP11* Mediates Reproduction in *Bemisia tabaci*

**DOI:** 10.3389/fphys.2020.00709

**Published:** 2020-06-30

**Authors:** Yang Zeng, Austin Merchant, Qingjun Wu, Shaoli Wang, Lan Kong, Xuguo Zhou, Wen Xie, Youjun Zhang

**Affiliations:** ^1^Department of Plant Protection, Institute of Vegetables and Flowers, Chinese Academy of Agricultural Sciences, Beijing, China; ^2^Department of Entomology, University of Kentucky, Lexington, KY, United States; ^3^Department of Computer Science, Eastern Kentucky University, Richmond, KY, United States

**Keywords:** chemosensory proteins, *Bemisia tabaci*, RNA interference, expression profiles, reproduction

## Abstract

The olfactory system serves a vital role in the evolution and survival of insects, being involved in behaviors such as host seeking, foraging, mating, and oviposition. Odorant-binding proteins (OBPs) and chemosensory proteins (CSPs) are involved in the olfactory recognition process. In this study, *BtabCSP11*, a *CSP11* gene from the whitefly *Bemisia tabaci*, was cloned and characterized. The open reading frame of *BtabCSP11* encodes 136 amino acids, with four highly conserved cysteine residues. The temporal and spatial expression profiles showed that *BtabCSP11* was highly expressed in the abdomens of *B. tabaci* females. Dietary RNA interference (RNAi)-based functional analysis showed substantially reduced fecundity in parthenogenetically reproduced females, suggesting a potential role of *BtabCSP11* in *B. tabaci* reproduction. These combined results expand the function of CSPs beyond chemosensation.

## Introduction

The insect olfactory system is used extensively in a variety of contexts, such as during host seeking, foraging, mating, and oviposition behaviors. Odorant-binding proteins (OBPs) and chemosensory proteins (CSPs) are involved in the olfactory recognition process (Pelosi et al., 2014, 2018). Both OBPs and CSPs are globular water-soluble acidic proteins with low isoelectric points found at high concentrations surrounding the chemosensory neurons (Pelosi et al., 2006). However, CSPs share minimal sequence similarity with OBPs. Generally, CSPs appear to be more conserved and are usually smaller (10–15 kDa) than OBPs (15–17 kDa). All CSPs possess four conserved cysteines (Pelosi et al., 2018). Previous studies have shown that CSPs are widely distributed in chemosensory organs, excluding the antennae (Maleszka and Stange, 1997; Nagnan-Le Meillour et al., 2000; Jin et al., 2005). In addition, CSPs are broadly expressed in non-chemosensory tissue, such as the subcuticular layer and wings (Marchese et al., 2000; Ban et al., 2003; Lu et al., 2007). The ubiquitous expression of CSPs suggests that, in addition to their known role in chemosensation, CSPs are involved in other physiological functions. In the honeybee, *Apis mellifera*, and the diamondback moth, *Plutella xylostella*, CSPs play a role in embryonic development (Maleszka et al., 2007; Gong et al., 2010). In addition, CSPs are involved in insecticide resistance in the whitefly *Bemisia. tabaci* and the mosquito *Anopheles gambiae* (Liu et al., 2014, 2016; Ingham et al., 2019). CSP3 knockdown reduced female survival and reproduction in the beet armyworm, *Spodoptera exigua* (Gong et al., 2012). Pheromone production, sexual behavior, mating, ovulation and oviposition are the main reproductive events. These events play roles in regulation of reproduction in insects (Raabe, 1987). Several studies have reported that insect CSPs can bind sex pheromone analogs and involved in reproduction (Dani et al., 2011; Iovinella et al., 2013).

The whitefly species *Bemisia tabaci*, one of the world’s most invasive agricultural pests, causes substantial crop losses worldwide by feeding on phloem and transmitting plant viruses. *Bemisia* Middle East-Asia Minor1 (MEAM1 or “B”) and Mediterranean (MED or “Q”) are the two most invasive biotypes, and have invaded nearly 60 countries in the past two decades (De Barro et al., 2011; Gilbertson et al., 2015; Wan and Yang, 2016). Because of its short life cycle and high fecundity, whitefly management has been extremely challenging. Whitefly control has relied predominantly on synthetic insecticides. Due to overuse, *B. tabaci* has developed resistance to most commercially available insecticides, particularly the neonicotinoids (Elbert and Nauen, 2000; Horowitz et al., 2004; Wang et al., 2010; Zheng et al., 2017). Therefore, new control alternatives, such as RNA interference (RNAi), are urgently needed.

RNAi has shown potential as a viable control alternative for agricultural pests (Upadhyay et al., 2011). Baum et al. showed a significant reduction in root damage caused by the Western corn rootworm, *Diabrotica virgifera virgifera*, by feeding larvae insecticidal dsRNAs in a growth chamber assay (Baum et al., 2007). In the meantime, Mao et al. (2007) demonstrated that larval growth in the cotton bollworm, *Helicoverpa armigera*, was arrested when fed transgenic leaves containing dsRNA corresponding to a detoxification enzyme gene. Both of these studies suggest that RNAi can be exploited to control insect pests. Similarly, studies on *B. tabaci* suggest that this biotechnology has potential in controlling them as well (Ghanim et al., 2007; Upadhyay et al., 2011).

Prior research has documented CSPs in the *B. tabaci* MEAM1 and MED biotypes (Li et al., 2012; Liu et al., 2016; Wang et al., 2017; Zeng et al., 2019). Functional analyses, however, are limited. In this study, we explored the function of *BtabCSP11* in *B. tabaci.* Based on previous research, we hypothesized that *BtabCSP11* is associated with reproductive behavior in *B. tabaci.* To test this hypothesis, we carried out the following experiments: (1) we cloned and analyzed the structure of *BtabCSP11*, and constructed a phylogenetic tree to analyze its evolutionary relationship with other insect CSPs; (2) we measured the temporospatial distribution of *BtabCSP11* throughout different developmental stages and across different tissue types; and finally (3) we investigated the function of *BtabCSP11* using dietary RNAi.

## Materials and Methods

### *Bemisia tabaci* Maintenance and Sample Collection

*Bemisia tabaci* MED populations were maintained on cotton plants at 27 ± 1°C under a L:D 16:8 photoperiod and 70 ± 10% relative humidity (RH). The identities of these strains were monitored every three generations using a mitochondrial marker, cytochrome oxidase I (mtCO I) (Chu et al., 2010). Different developmental stages, including eggs, the four nymphal stages, and adult females and males; and adult tissue types, including head, abdomen and a mixture of thorax, legs and wings, were collected separately from three *B. tabaci* MED populations. A total of 500 *B. tabaci* were collected per replicate for the egg and four nymphal stages, while 100 individuals were collected per replicate for adult females and males. Three independent biological replicates were used for each sample. Samples were rapidly frozen in liquid nitrogen and stored at -80°C for the subsequent RT-qPCR analyses.

### RNA Extraction, cDNA Synthesis, and Molecular Cloning of *BtabCSP11*

Total RNA was extracted using TRIzol reagent (Invitrogen, Carlsbad, CA, USA) according to the manufacturer’s instructions. RNA was quantified using a NanoDrop 2000 (Thermo Scientific, Wilmington, DE, United States), and integrity was checked with 1% Tris/borate/EDTA (TBE) agarose gel electrophoresis. For gene cloning and RT-qPCR analysis, first-strand cDNA was synthesized using 1 μg of total RNA with the PrimeScript^®^ RT reagent kit (TaKaRa Biotech, Kyoto, Japan) following the manufacturer’s recommendations. The synthesized first-strand cDNA was either used immediately or stored at −20°C for later use. We obtained the full-length sequence of *BtabCSP11* from the *B. tabaci* MED genome and transcriptome (Xie et al., 2017).

### Sequence Analysis

The program SignalPV5.0^1^ was used to predict the putative N-terminal signal peptides and cleavage sites of *BtabCSP11*. The occurrence of α-helices and molecular weight were predicted using ExPASy^2^. The CSPs of other insect species discussed in this study were retrieved from the NCBI database. ClustalW was used to align the sequences with default gap penalty parameters of gap opening = 10 and extension = 0.2. An alignment graph was generated using WebLogo.

### Phylogenetic Analysis

A neighbor-joining tree was constructed using MEGA 6.0 with a *p*-distance model and pairwise deletion of gaps (Tamura et al., 2013). The bootstrap support of tree branches was assessed by resampling amino acid positions 1,000 times. Sequences used in the phylogenetic analysis were open reading frames (Supplementary Table S1). Phylogenetic trees were then presented in circular shape and colored taxonomically using online tools provided by Evolview (He et al., 2016).

### Expression Profiles of *BtabCSP11*

Expression profiles of *BtabCSP11* throughout different developmental stages of *B. tabaci* MED were obtained using transcriptome data (SRP064690). In addition, we validated transcriptomic profiles using RT-qPCR analysis (Zeng et al., 2019). Five two-fold serial dilutions of whitefly cDNA template were used to determine RT-qPCR primer amplification efficiencies through dissociation curve analysis. Only primers with 90–110% amplification efficiencies were used for subsequent analysis. Relative quantification was calculated using the 2^–Δ^ ^Δ^ ^Ct^ method, and mRNA expression values were normalized to the recommended reference genes *EF1-*α, *SDHA* and *Actin* (Li et al., 2013). Three biological and four technical replicates were used for each sample. Significant differences between samples were determined using one-way ANOVA with Tukey’s HSD test. SPSS20.0 was used to analyze correlations between RT-qPCR and RNA-seq data.

### Dietary RNAi

dsRNA primers of *BtabCSP11* and *EGFP* (GenBank: KC896843) with a T7 promoter sequence were designed using Primer Premier 5.0 (Table 1). dsRNAs of *BtabCSP11* and *EGFP* were synthesized using the T7 Ribomax^TM^ Express RNAi System (Promega, Madison, WI, United States). The quality of dsRNA was evaluated by gel electrophoresis, and dsRNA concentration was quantified using a NanoDrop spectrophotometer.

**TABLE 1:** Primers used for this study.

Primer Name	Anneal Temp (°C)	Primer Sequence (5′–3′)
**RACE PCR**		
BtabCSP11-F	58	CGTTTGGGCGTCTTGATG
BtabCSP11-R	58	GCAACTCAGACCGGGGAC
**RT-qPCR**		
BtabCSP11-F-RT	60	GTCCTTGCACTAACGAGGGG
BtabCSP11-R-RT	60	AACTGTGCGCACTATCCTCC
EF-1a-F	60	TAGCCTTGTGCCAATTTCCG
EF-1a-R	60	CCTTCAGCATTACCGTCC
Actin-F	60	TCTTCCAGCCATCCTTCTTG
Actin-R	60	CGGTGATTTCCTTCTGCATT
SDHA-F	60	GCGACTGATTCTTCTCCTGC
SDHA-R	60	TGGTGCCAACAGATTAGGTGC
**Dietary RNAi**		
dsBtabCSP11-F	58	TAATACGACTCACTATAGGGA GATCCGCCATTAGTGATGATGA
dsBtabCSP11-R	58	TAATACGACTCACTATAGGGA GATGTCTTCGTCCATGAACTCG
dsEGFP-F	58	TAATACGACTCACTATAGGG TGAGCAAGGGCGAGGAG
dsEGFP-R	58	TAATACGACTCACTATAGGGCG GCGGTCACGAACTCCAG

*Anneal temp: annealing temperature.*

Knockdown of *BtabCSP11* was performed by orally feeding dsRNAs to *B. tabaci* MED adult females in a feeding chamber. The feeding chambers contained 200 μL of diet solution, which consisted of 30% sucrose, 5% yeast extract (weight/volume), ddH_2_O and 0.5 μg/μL dsBtabCSP11. Approximately 45 newly emerged (<2 days old) *B. tabaci* MED adult females were released into the feeding chambers, and were kept at 25°C, 80% RH, and a L:D 16:8 photoperiod. The effectiveness of RNAi was evaluated by RT-qPCR 2 days post-feeding. Each RNAi treatment was repeated six times.

### Oviposition Bioassay

After feeding, fecundity (Berger et al., 2008), i.e., the number of eggs laid per adult female, was counted at day 1, 3, 7, and 10. Fresh cotton leaves were provided every day. *Bemisia tabaci* reproduction was recorded as the mean number of eggs per surviving whitefly laid. A total of six independent biological replicates were used for each sample. Data were analyzed with SPSS20.0. Differences among treatments were evaluated using one-way ANOVA with Tukey’s HSD test. Figures were generated using SigmaPlot 12.5.

## Results

### Identification and Sequence Analysis of *BtabCSP11*

**FIGURE 1:**
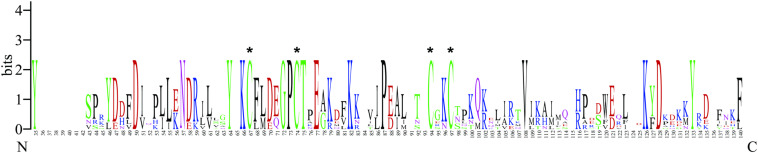
WebLogo alignment of *BtabCSP11* with six other hemipteran CSPs. “*”denotes highly conserved cysteines. N, N-terminus, C, C-terminus.

The full-length cDNA of *BtabCSP11* contains a 408 bp open reading frame (ORF) encoding 136 amino acids (GenBank number: XP_018916537.1) with a 16.2 kDa molecular weight. There is a signal peptide with 18 residues at the N-terminus of *BtabCSP11*. Consistent with a classical model Cys-X6-8-Cys-X16-21-Cys-X2-4-Cys, *BtabCSP11* has four conserved cysteine residues and six α-helices (Figure 1 and Supplementary Figure S1).

**FIGURE 2:**
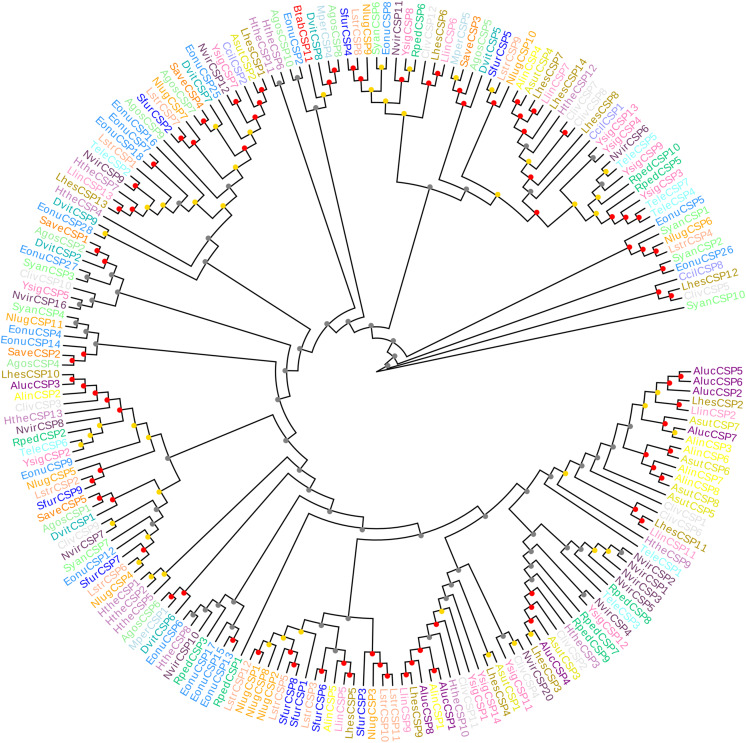
Neighbor-joining phylogenetic tree comparing *BtabCSP11* with known hemipteran CSPs. Species were grouped by different colors. Bootstrap values were calculated with 1,000 replications, and bootstraps were marked on the nodes (0–40 gray, 40–80 yellow, 80–100 red). The protein names and sequences of the 200 CSPs used in this analysis are listed in Supplementary Table S2. Alin = *Adelphocoris lineolatus*, Agos = *Aphis gossypii*, Aluc = *Apolygus lucorum*, Mper = *Myzus persicae*, Nlug = *Nilaparvata lugens*, Sfur = *Sogatella furcifera*, Ccil = *Corythucha ciliata*, Syan = *Subpsaltria yangi*, Asut = *Adelphocoris suturalis*, Lhes = *Lygus Hesperus*, Save = *Sitobion avenae*, Lstr = *Laodelphax striatellus*, Nvir = *Nezara viridula*, Hthe = *Helopeltis theivora*, Ysig = *Yemma signatus*, Cliv = *Cyrtorhinus lividipennis*, Eonu = *Empoasca onukii*, Tele = *Tropidothorax elegans*, Rped = *Riptortus pedestris*, Llin = *Lygus lineolaris*, Dvit = *Daktulosphaira vitifoliae* and Btab = *Bemsia tabaci.*

The CSP11 genes of other insect species were chosen for multiple sequence alignment with *BtabCSP11* (Supplementary Table S1). A total of seven CSPs with >40% sequence similarity with *BtabCSP11* (Pelosi et al., 2018), including *DhouCSP, DvitCSP8, DkikCSP, CmedCSP3, PrapCSP10*, and *CbowCSP7*, were included in the WebLogo alignment (Figure 1). The results of the alignment showed four highly conserved cysteine residues at positions 67, 74, 94, and 97; other positions such as 35 (Y), 50 (D), 57 (N), 64 (Y), 66 (K), 72 (G), 73 (P), 75 (T), 77 (E), 82 (K), 87 (P), 108 (V), 126 (K), 128 (D), and 133 (Y) were also highly conserved. The complete sequence alignment including all insect CSP11s is displayed in Supplementary Figure S1. Phylogenetic analysis indicated that the hemipteran CSPs formed four large branches, of which *BtabCSP11, MperCSP4, DvitCSP8*, and *AgosCSP8* were clustered into one group (Figure 2).

### Temporospatial Expression Profiles of *BtabCSP11*

**FIGURE 3:**
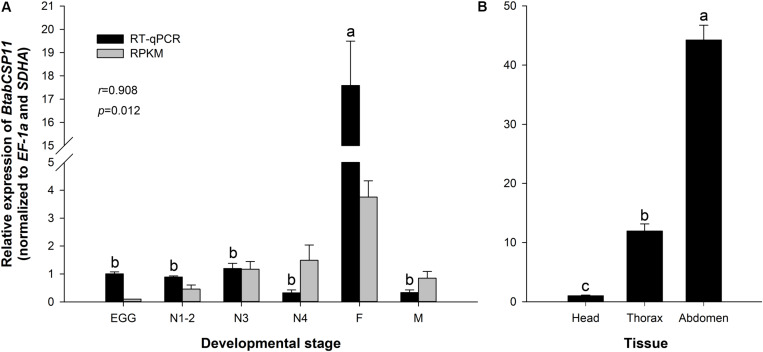
Temporospatial expression profiles of *BtabCSP11*. **(A)** Expression analysis of the *BtabCSP11* gene by RT-qPCR (black bars) and RNA-seq (black lines). E = egg, N = nymph stages 1–4 as indicated, F = adult female, and M = adult male. **(B)** *B. tabaci BtabCSP11* gene transcript levels in different tissue types. Standard error for each sample is represented by error bar and different letters (a, b, c) above each bar denote significant differences (*P* < 0.05).

The transcriptomic profiles of *BtabCSP11* were confirmed by RT-qPCR analysis (*P* < 0.05 *r* = 0.908, Figure 3A). Among different developmental stages, *BtabCSP11* expression was significantly higher in adult females (*F* = 76.988, *P* < 0.0001), and there were no significant differences among the remaining developmental stages (Figure 3A). Among different tissue types, *BtabCSP11* expression was significantly higher in abdomen tissue than in head and thorax tissue (Figure 3B).

### Functional Analysis of *BtabCSP11*

**FIGURE 4:**
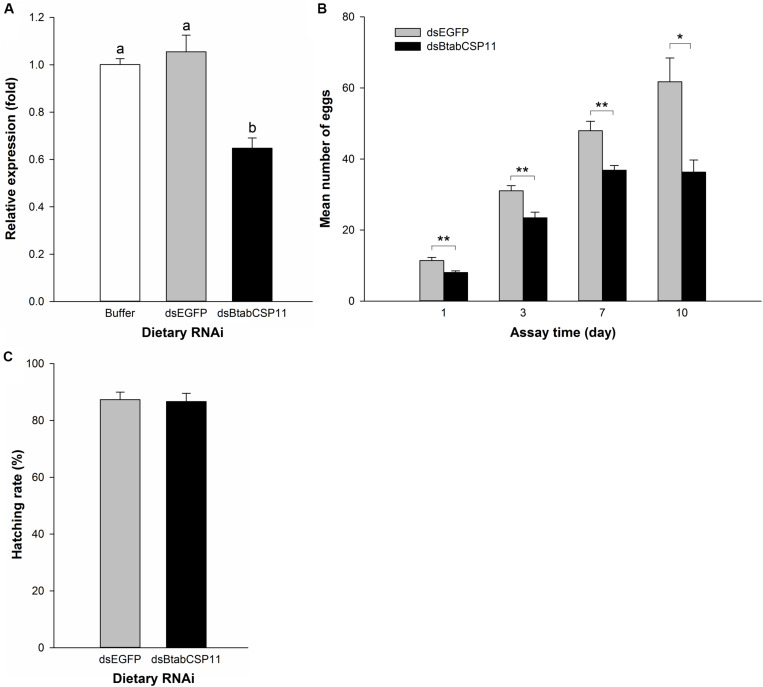
Dietary RNAi-based functional analysis of *BtabCSP11* **(A)** Silencing of *BtabCSP11* gene expression by dietary RNAi. Suppression of *BtabCSP11* expression after *B. tabaci* MED adult females fed on dsRNA for 48 h. Expression of the *BtabCSP11* gene was detected by RT-qPCR with *EF1 –*α and *actin* as the reference genes. Different letters indicate significant differences between treatments (*p* < 0.05; *n* = 3). **(B)** Effect of *BtabCSP11* silencing on *B. tabaci* female reproduction. Values marked with “*” are significantly different based on one-way analysis of variance (ANOVA with Tukey’s HSD test). **(C)** Hatching rate.

To explore the function of *BtabCSP11* in *B. tabaci*, we silenced this gene using dietary RNAi. In comparison to control groups, *BtabCSP11* expression in *dsBtabCSP11*-treated groups was suppressed significantly at 48 h post-feeding (Figure 4A). The total number of eggs laid by *dsBtabCSP11* females at days 1, 3, 7, and 10 post-feeding was significantly lower than that in controls (*P* < 0.05; Figure 4B). There was no significant difference in hatching rate between the treatment (*dsBtabCSP11*) and control (*dsEGFP*) groups (Figure 4C).

## Discussion

In this study, we analyzed the genetic sequence of *BtabCSP11* in *B. tabaci* and found that *BtabCSP11* has four conserved cysteines and six helices connected by α-α loops (Wanner et al., 2004). BtabCSP11 displayed about 40% sequence similarity to the CSPs of phylogenetically distant species such as the cabbage beetle *Colaphellus bowringi* (Pelosi et al., 2005, 2018).

Kulmuni and Havukainen (2013) showed that 5-helical CSPs are the only highly conserved CSPs in Arthropoda and are likely involved in functions other than chemosensation, whereas the vastly divergent 6-helical CSPs carry out solely chemosensory functions. Evidence strongly suggests that CSPs might be involved in chemodetection, as are OBPs (Waris et al., 2018, 2020b). In the honeybee, CSP3 specifically binds some components of brood pheromone (Briand et al., 2002). In the paper wasp *Polistes dominulus* (Calvello et al., 2003), the tsetse fly *Glossina morsitans morsitans* (Liu et al., 2012), and several species of ants, some CSPs are specifically expressed in antennae (McKenzie et al., 2014) and have been proposed to be associated with host-seeking behavior. In the plant bug *Adelphocoris lineolatus*, three CSPs have high binding affinity with host-related chemicals (Gu et al., 2012). Similarly, SinfCSP19 plays a role in the reception of host plant volatiles by the stem borer *Sesamia inferens* (Zhang et al., 2014). In the planthopper *Nilaparvata lugens*, NlugCSP10 may detect volatiles emitted from host plants (Waris et al., 2020a). In addition to host plant volatiles, study has reported that insect CSPs can bind β-carotene (Zhu et al., 2016).

CSPs are widely expressed in the head, thorax, abdomen, legs, wings, testes and ovaries (Gong et al., 2007; Lu et al., 2007). This expression pattern indicates that CSPs may be involved in a variety of physiological processes. Previous reports show that CSPs have many functions outside of simply transporting and binding odor molecules. For example, studies have shown that CSPs are involved in the growth and development of honeybees, the molting process of ant larvae, the reproduction of the beet armyworm *S. exigua*, and insecticide resistance in the mosquito *A. gambiae* (Maleszka et al., 2007; Gong et al., 2012; Pelosi et al., 2018; Ingham et al., 2019). Inhibition of CSP9 expression by RNAi affects fatty acid biosynthesis and prevents cuticle development in the fire ant *Solenopsis invicta* (Cheng et al., 2015). In addition, some CSPs act as carriers of visual pigments, as in the cotton bollworm, *Helicoverpa armigera* (Zhu et al., 2016). CSPs have also been found to play roles in the reproductive processes of insects, such that a decrease in CSP gene expression negatively affects reproduction (Gong et al., 2012; Ma et al., 2019).

Given that phylogenetic analysis clustered *BtabCSP11* with *MperCSP4, DvitCSP8*, and *AgosCSP8*, additional functional studies are warranted to resolve the evolutionary relationship within this group. This kind of evolutionary relationship also occurs among other insects (Vieira and Rozas, 2011; Eyun et al., 2017). Additional functional studies should be carried out to understand the evolutionary history of CSPs in Hemiptera.

Our temporospatial distribution study demonstrated that *BtabCSP11* was highly expressed in the abdomens of adult females, suggesting a potential role in reproduction. Our dietary RNAi-based functional study confirmed that silencing of *BtabCSP11* significantly reduced oviposition in *B. tabaci* (Figure 4). Similarly, Gong et al. (2012) reported that silencing of CSP3 in *S. exigua* led to a 71.4% reduction in oviposition in comparison to uninjected controls. In addition, silencing of CSP12 in the leaf beetle *Ophraella communa* resulted in a 28% reduction in the number of eggs laid (Ma et al., 2019). Potential causes for this change include altered reproductive behavior (e.g., mating and oviposition). In this study, however, males were not included and females were not provided a choice of oviposition site. Therefore, we hypothesize that *BtabCSP11* may affect the oviposition behavior of *B. tabaci.*

## Data Availability Statement

All datasets generated for this study are included in the article/Supplementary Material.

## Author Contributions

The research was designed by WX and YJZ. The experiments were conceived by YZ. Data was analyzed by YZ, LK, and XZ. Manuscript was drafted by YZ. Manuscript was revised and finalized by WX, XZ, AM, SW, QW, LK, and YJZ. All authors contributed to the article and approved the submitted version.

## Conflict of Interest

The authors declare that the research was conducted in the absence of any commercial or financial relationships that could be construed as a potential conflict of interest.

## Footnotes

1 http://www.cbs.dtu.dk/services/SignalP/

2 https://www.expasy.org/
