# Supplementary material for: A Chemosensory Protein BtabCSP11 Mediates Reproduction in Bemisia tabaci
Source: Front Physiol. 2020 Jun 30;11:709. doi: 10.3389/fphys.2020.00709 (PMC7338578; doi:10.3389/fphys.2020.00709)
Supplement: Supplementary file 1 [file Table_1.DOCX]

**Supplementary Materials**

**Supplementary Figure**

**Figure S1**. **Alignment of BtabCSP11 with other Hemipterans.** The four conserved cysteine residues are marked with an asterisk. The six predicted α -helices are indicated by spiral lines. Dple = *Danaus plexippus plexippus*, Csin = *Conopomorpha sinensis*, Agra = *Anthonomus grandis*, Lhes = *Lygus hesperus*, Llin = *Lygus lineolaris*, Cpin = *Conogethes pinicolalis*, Msep = *Mythimna separate*, Ysig = *Yemma signatus*, Cliv = *Cyrtorhinus lividipennis*, Eobl = *Ectropis oblique*, Cnip = *Chrysoperla nipponensis*, Cpal = *Chrysopa pallens*, Nlug = *Nilaparvata lugens*, Harm = *Helicoverpa armigera*, Tcas = *Tribolium castaneum*, Gmel = *Galleria mellonella*, Lstr = *Laodelphax striatellus*, Prap = *Pieris rapae*, Oasi = *Oedaleus asiaticus*, Malt = *Monochamus alternatus*, Sexi = *Spodoptera exigua*, Nvir = *Nezara viridula*, Oinf = *Oedaleus infernalis*, Csas = *Carposina sasakii*, Dpun = *Dendrolimus punctatus*, Ofur = *Ostrinia furnacalis*, Cbow = *Colaphellus bowringi*, Gmol = *Grapholita molesta*, Dpon = *Dendroctonus ponderosae*, Bmor = *Bombyx mori*, Ccun = *Chouioia cunea* and Btab = *Bemsia tabaci.*

**Supplementary Table**

**Table S1.** **Sequences used in the WebLogo analysis and sequence alignment.**

| **Species** | **Order** | **Sequence Name** | **Accession Number** | **Sequence Length** |
| --- | --- | --- | --- | --- |
| *WebLogo analysis* | | | | |
| *Daktulosphaira vitifoliae* | Hemiptera | DvitCSP8 | ARO50012.1 | 142 |
| *Colaphellus bowringi* | Coleoptera | CbowCSP7 | ALR72521.1 | 125 |
| *Dendrolimus kikuchii* | Lepidoptera | DkikCSP | AII01036.1 | 123 |
| *Cnaphalocrocis medinalis* | Lepidoptera | CmedCSP3 | AGI37365.1 | 123 |
| *Pieris rapae* | Lepidoptera | PrapCSP10 | QDW65476.1 | 123 |
| *Dendrolimus houi* | Lepidoptera | DhouCSP | AII01020.1 | 123 |
| *Sequence alignment (Figure S1)* | | | | |
| *Chrysoperla nipponensis* | Neuroptera | CnipCSP11 | AKW47214.1 | 106 |
| *Chrysopa pallens* | Neuroptera | CpalCSP11 | AKW47183.1 | 110 |
| *Colaphellus bowringi* | Coleoptera | CbowCSP11 | ALR72525.1 | 113 |
| *Anthonomus grandis* | Coleoptera | AgraCSP11 | AVI04881.1 | 120 |
| *Tribolium castaneum* | Coleoptera | TcasCSP11 | EFA07563.1 | 133 |
| *Monochamus alternatus* | Coleoptera | MaltCSP11 | AIX97086.1 | 125 |
| *Dendroctonus ponderosae* | Coleoptera | DponCSP11 | AGI05163.1 | 102 |
| *Helicoverpa armigera* | Lepidoptera | HarmCSP11 | AFR92095.1 | 128 |
| *Conopomorpha sinensis* | Lepidoptera | CsinCSP11 | QGN03660.1 | 123 |
| *Galleria mellonella* | Lepidoptera | GmelCSP11 | QEI46809.1 | 126 |
| *Carposina sasakii* | Lepidoptera | CsasCSP11 | AYD42215.1 | 133 |
| *Ostrinia furnacalis* | Lepidoptera | OfurCSP11 | BAV56815.1 | 145 |
| *Grapholita molesta* | Lepidoptera | GmolCSP11 | ALC79597.1 | 123 |
| *Bombyx mori* | Lepidoptera | BmorCSP11 | ABH88204.1 | 121 |
| *Danaus plexippus plexippus* | Lepidoptera | DpleCSP11 | OWR49803.1 | 122 |
| *Spodoptera exigua* | Lepidoptera | SexiCSP11 | AKT26487.1 | 122 |
| *Mythimna separate* | Lepidoptera | MsepCSP11 | AWT22258.1 | 120 |
| *Pieris rapae* | Lepidoptera | PrapCSP11 | QDW65477.1 | 116 |
| *Dendrolimus punctatus* | Lepidoptera | DpunCSP11 | ARO70315.1 | 122 |
| *Ectropis oblique* | Lepidoptera | EoblCSP11 | ALS03836.1 | 112 |
| *Conogethes pinicolalis* | Lepidoptera | CpinCSP11 | QFR36138.1 | 108 |
| *Oedaleus asiaticus* | Orthoptera | OasiCSP11 | ATI99850.1 | 130 |
| *Oedaleus infernalis* | Orthoptera | OinfCSP11 | AYN71360.1 | 132 |
| *Cyrtorhinus lividipennis* | Hemiptera | ClivCSP11 | AXU25090.1 | 127 |
| *Lygus hesperus* | Hemiptera | LhesCSP11 | APB88047.1 | 132 |
| *Lygus lineolaris* | Hemiptera | LlinCSP11 | APB88074.1 | 132 |
| *Yemma signatus* | Hemiptera | YsigCSP11 | AYN07339.1 | 125 |
| *Nilaparvata lugens* | Hemiptera | NlugCSP11 | ASL05052.1 | 126 |
| *Laodelphax striatellus* | Hemiptera | LstrCSP11 | AGZ04939.1 | 103 |
| *Nezara viridula* | Hemiptera | NvirCSP11 | QCZ25125.1 | 127 |
| *Chouioia cunea* | Hymenoptera | CcunCSP11 | QGW50258.1 | 131 |

**Table S2.** **Sequences used in the phylogenetic analysis**

| **Species** | **Sequence Name** | **Accession Number** | **Sequence Length** | **References** |
| --- | --- | --- | --- | --- |
| *Myzus persicae* | MperCSP1 | ACJ64047.1 | 131 | Xu et al., 2009 |
|  | MperCSP4 | ACJ64048.1 | 163 |  |
|  | MperCSP5 | ACJ64049.1 | 139 |  |
| *Aphis gossypii* | AgosCSP1 | AGE97641.1 | 170 | Gu et al., 2013 |
|  | AgosCSP2 | AGE97642.1 | 134 |  |
|  | AgosCSP4 | AGE97643.1 | 145 |  |
|  | AgosCSP5 | AGE97644.1 | 139 |  |
|  | AgosCSP6 | AGE97645.1 | 131 |  |
|  | AgosCSP7 | AGE97646.1 | 152 |  |
|  | AgosCSP8 | AGE97647.1 | 162 |  |
|  | AgosCSP9 | AGE97648.1 | 171 |  |
|  | AgosCSP10 | AGE97649.1 | 149 |  |
| *Apolygus lucorum* | AlucCSP1 | AGD80081.1 | 130 |  |
|  | AlucCSP2 | AGD80082.1 | 132 |  |
|  | AlucCSP3 | AGD80083.1 | 123 |  |
|  | AlucCSP4 | AGD80084.1 | 128 |  |
|  | AlucCSP5 | AGD80085.1 | 130 |  |
|  | AlucCSP6 | AGD80086.1 | 132 |  |
|  | AlucCSP7 | AGD80087.1 | 132 |  |
|  | AlucCSP8 | AGD80088.1 | 124 |  |
| *Adelphocoris lineolatus* | AlinCSP1 | ACZ58019.1 | 130 | Gu et al., 2012 |
|  | AlinCSP2 | ACZ58021.1 | 123 |  |
|  | AlinCSP3 | ACZ58020.1 | 132 |  |
|  | AlinCSP4 | ACZ58022.1 | 127 |  |
|  | AlinCSP5 | ACZ58023.1 | 127 |  |
|  | AlinCSP6 | ACZ58024.1 | 132 |  |
|  | AlinCSP7 | ACZ58025.1 | 132 |  |
|  | AlinCSP8 | ACZ58026.1 | 132 |  |
| *Sogatella furcifera* | SfurCSP1 | AGZ04911.1 | 131 |  |
|  | SfurCSP2 | AGZ04912.1 | 112 |  |
|  | SfurCSP3 | AGZ04913.1 | 126 |  |
|  | SfurCSP4 | AGZ04914.1 | 177 |  |
|  | SfurCSP5 | AGZ04915.1 | 145 |  |
|  | SfurCSP6 | AGZ04916.1 | 128 |  |
|  | SfurCSP7 | AGZ04917.1 | 170 |  |
|  | SfurCSP8 | AGZ04918.1 | 130 |  |
|  | SfurCSP9 | AGZ04919.1 | 123 |  |
| *Nilaparvata lugens* | NlugCSP1 | ASL05006.1 | 131 |  |
|  | NlugCSP2 | ASL05045.1 | 132 |  |
|  | NlugCSP3 | ASL05046.1 | 129 |  |
|  | NlugCSP4 | ASL05047.1 | 171 |  |
|  | NlugCSP5 | ASL04982.1 | 124 |  |
|  | NlugCSP6 | ASL05030.1 | 126 |  |
|  | NlugCSP7 | ASL05048.1 | 114 |  |
|  | NlugCSP8 | ASL05049.1 | 129 |  |
|  | NlugCSP9 | ASL05050.1 | 189 |  |
|  | NlugCSP10 | ASL05051.1 | 129 |  |
|  | NlugCSP11 | ASL05052.1 | 126 |  |
| *Corythucha ciliata* | CcilCSP1 | ARI71706.1 | 125 |  |
|  | CcilCSP2 | ARW29607.1 | 111 |  |
|  | CcilCSP8 | ARW29608.1 | 133 |  |
| *Subpsaltria yangi* | SyanCSP1 | AXY87870.1 | 126 | Qi et al., 2018 |
|  | SyanCSP2 | AXY87871.1 | 115 |  |
|  | SyanCSP3 | AXY87872.1 | 196 |  |
|  | SyanCSP4 | AXY87873.1 | 143 |  |
|  | SyanCSP6 | AXY87875.1 | 130 |  |
|  | SyanCSP7 | AXY87876.1 | 126 |  |
|  | SyanCSP10 | AXY87879.1 | 137 |  |
| *Adelphocoris suturalis* | AsutCSP1 | ANA10243 | 123 | Cui et al., 2017 |
|  | AsutCSP2 | ANA10244.1 | 132 |  |
|  | AsutCSP3 | ANA10245.1 | 128 |  |
|  | AsutCSP4 | ANA10246.1 | 127 |  |
|  | AsutCSP5 | ANA10247.1 | 131 |  |
|  | AsutCSP6 | ANA10248.1 | 132 |  |
|  | AsutCSP7 | ANA10249.1 | 132 |  |
|  | AsutCSP8 | ANA10250.1 | 132 |  |
| *Lygus Hesperus* | LhesCSP1 | APB88037.1 | 111 |  |
|  | LhesCSP2 | APB88038.1 | 135 |  |
|  | LhesCSP3 | APB88039.1 | 128 |  |
|  | LhesCSP4 | APB88040.1 | 130 |  |
|  | LhesCSP5 | APB88041.1 | 133 |  |
|  | LhesCSP6 | APB88042.1 | 196 |  |
|  | LhesCSP7 | APB88043.1 | 127 |  |
|  | LhesCSP8 | APB88044.1 | 123 |  |
|  | LhesCSP9 | APB88045.1 | 130 |  |
|  | LhesCSP10 | APB88046.1 | 123 |  |
|  | LhesCSP11 | APB88047.1 | 132 |  |
|  | LhesCSP12 | APB88048.1 | 135 |  |
|  | LhesCSP13 | APB88051.1 | 113 |  |
|  | LhesCSP14 | APB88063.1 | 126 |  |
| *Sitobion avenae* | SaveCSP1 | AFD20366.1 | 123 | Xue et al., 2016 |
|  | SaveCSP2 | APB03438.1 | 146 |  |
|  | SaveCSP3 | APB03439.1 | 137 |  |
|  | SaveCSP4 | APB03440.1 | 156 |  |
|  | SaveCSP5 | APB03441.1 | 230 |  |
| *Laodelphax striatellus* | LstrCSP1 | AGZ04929.1 | 110 |  |
|  | LstrCSP2 | AGZ04930.1 | 123 |  |
|  | LstrCSP3 | AGZ04931.1 | 127 |  |
|  | LstrCSP4 | AGZ04932.1 | 126 |  |
|  | LstrCSP5 | AGZ04933.1 | 132 |  |
|  | LstrCSP6 | AGZ04934.1 | 174 |  |
|  | LstrCSP7 | AGZ04935.1 | 139 |  |
|  | LstrCSP8 | AGZ04936.1 | 178 |  |
|  | LstrCSP9 | AGZ04937.1 | 148 |  |
|  | LstrCSP10 | AGZ04938.1 | 112 |  |
|  | LstrCSP11 | AGZ04939.1 | 103 |  |
|  | LstrCSP12 | AGZ04940.1 | 130 |  |
| *Nezara viridula* | NvirCSP1 | QCZ25115.1 | 126 |  |
|  | NvirCSP2 | QCZ25116.1 | 135 |  |
|  | NvirCSP3 | QCZ25117.1 | 128 |  |
|  | NvirCSP4 | QCZ25118.1 | 117 |  |
|  | NvirCSP5 | QCZ25119.1 | 126 |  |
|  | NvirCSP6 | QCZ25120.1 | 134 |  |
|  | NvirCSP7 | QCZ25121.1 | 124 |  |
|  | NvirCSP8 | QCZ25122.1 | 126 |  |
|  | NvirCSP9 | QCZ25123.1 | 109 |  |
|  | NvirCSP10 | QCZ25124.1 | 127 |  |
|  | NvirCSP11 | QCZ25125.1 | 127 |  |
|  | NvirCSP12 | QCZ25126.1 | 109 |  |
|  | NvirCSP16 | QCZ25130.1 | 134 |  |
|  | NvirCSP20 | QCZ25133.1 | 116 |  |
| *Helopeltis theivora* | HtheCSP1 | QCX43081.1 | 162 |  |
|  | HtheCSP2 | QCX43082.1 | 128 |  |
|  | HtheCSP3 | QCX43083.1 | 129 |  |
|  | HtheCSP4 | QCX43084.1 | 109 |  |
|  | HtheCSP5 | QCX43085.1 | 131 |  |
|  | HtheCSP6 | QCX43086.1 | 120 |  |
|  | HtheCSP7 | QCX43087.1 | 120 |  |
|  | HtheCSP8 | QCX43088.1 | 129 |  |
|  | HtheCSP9 | QCX43089.1 | 134 |  |
|  | HtheCSP10 | QCX43090.1 | 128 |  |
|  | HtheCSP11 | QCX43091.1 | 124 |  |
|  | HtheCSP12 | QCX43092.1 | 129 |  |
|  | HtheCSP13 | QCX43093.1 | 121 |  |
| *Yemma signatus* | YsigCSP1 | AYN07329.1 | 131 |  |
|  | YsigCSP2 | AYN07330.1 | 131 |  |
|  | YsigCSP3 | AYN07331.1 | 140 |  |
|  | YsigCSP4 | AYN07332.1 | 137 |  |
|  | YsigCSP5 | AYN07333.1 | 129 |  |
|  | YsigCSP7 | AYN07335.1 | 106 |  |
|  | YsigCSP8 | AYN07336.1 | 145 |  |
|  | YsigCSP9 | AYN07337.1 | 135 |  |
|  | YsigCSP11 | AYN07339.1 | 125 |  |
|  | YsigCSP12 | AYN07340.1 | 140 |  |
|  | YsigCSP13 | AYN07341.1 | 132 |  |
|  | YsigCSP14 | AYN07342.1 | 118 |  |
| *Cyrtorhinus lividipennis* | ClivCSP1 | ARJ35776.1 | 135 |  |
|  | ClivCSP2 | ARJ35777.1 | 145 |  |
|  | ClivCSP3 | ARJ35778.1 | 121 |  |
|  | ClivCSP4 | ARJ35779.1 | 151 |  |
|  | ClivCSP5 | ARJ35780.1 | 126 |  |
|  | ClivCSP6 | AXU25085.1 | 131 |  |
|  | ClivCSP7 | AXU25086.1 | 126 |  |
|  | ClivCSP8 | AXU25087.1 | 127 |  |
|  | ClivCSP9 | AXU25088.1 | 141 |  |
|  | ClivCSP10 | AXU25089.1 | 120 |  |
|  | ClivCSP11 | AXU25090.1 | 127 |  |
|  | ClivCSP12 | AXU25091.1 | 157 |  |
| *Empoasca onukii* | EonuCSP2 | AWC68021.1 | 122 |  |
|  | EonuCSP4 | AWC68023.1 | 123 |  |
|  | EonuCSP5 | AWC68022.1 | 126 |  |
|  | EonuCSP6 | AWC68037.1 | 132 |  |
|  | EonuCSP7 | AWC68020.1 | 106 |  |
|  | EonuCSP8 | AWC68026.1 | 158 |  |
|  | EonuCSP9 | AWC68028.1 | 121 |  |
|  | EonuCSP12 | AWC68031.1 | 123 |  |
|  | EonuCSP13 | AWC68032.1 | 125 |  |
|  | EonuCSP14 | AWC68033.1 | 133 |  |
|  | EonuCSP15 | AWC68034.1 | 129 |  |
|  | EonuCSP16 | AWC68035.1 | 108 |  |
|  | EonuCSP17 | AWC68036.1 | 131 |  |
|  | EonuCSP18 | AWC68024.1 | 116 |  |
|  | EonuCSP25 | AWC68025.1 | 112 |  |
|  | EonuCSP26 | AWC68027.1 | 111 |  |
|  | EonuCSP27 | AWC68029.1 | 130 |  |
|  | EonuCSP28 | AWC68030.1 | 150 |  |
| *Tropidothorax elegans* | TeleCSP1 | AXB87335.1 | 134 | Sun et al., 2015 |
|  | TeleCSP2 | AXB87336.1 | 115 |  |
|  | TeleCSP3 | AXB87337.1 | 125 |  |
|  | TeleCSP4 | AXB87338.1 | 142 |  |
|  | TeleCSP5 | AXB87339.1 | 127 |  |
|  | TeleCSP6 | AXB87340.1 | 123 |  |
|  | TeleCSP7 | AXB87341.1 | 135 |  |
| *Riptortus pedestris* | RpedCSP1 | AWW17225.1 | 127 | Sun et al., 2015 |
|  | RpedCSP2 | AWW17226.1 | 125 |  |
|  | RpedCSP3 | AWW17227.1 | 133 |  |
|  | RpedCSP5 | AWW17229.1 | 133 |  |
|  | RpedCSP6 | AWW17230.1 | 130 |  |
|  | RpedCSP7 | AWW17231.1 | 126 |  |
|  | RpedCSP8 | AWW17232.1 | 130 |  |
|  | RpedCSP9 | AWW17233.1 | 132 |  |
|  | RpedCSP10 | AWW17234.1 | 131 |  |
| *Lygus lineolaris* | LlinCSP2 | APB88065.1 | 135 |  |
|  | LlinCSP5 | APB88068.1 | 133 |  |
|  | LlinCSP6 | APB88069.1 | 196 |  |
|  | LlinCSP7 | APB88070.1 | 127 |  |
|  | LlinCSP9 | APB88072.1 | 130 |  |
|  | LlinCSP11 | APB88074.1 | 132 |  |
|  | LlinCSP13 | APB88076.1 | 113 |  |
| *Daktulosphaira vitifoliae* | DvitCSP1 | ARO50007.1 | 249 |  |
|  | DvitCSP2 | ARO50008.1 | 150 |  |
|  | DvitCSP5 | ARO50009.1 | 133 |  |
|  | DvitCSP6 | ARO50010.1 | 132 |  |
|  | DvitCSP7 | ARO50011.1 | 145 |  |
|  | DvitCSP8 | ARO50012.1 | 142 |  |
|  | DvitCSP9 | ARO50013.1 | 207 |  |
